# Dynamic fracture mechanics and energy distribution rate response characteristics of coal containing bedding structure

**DOI:** 10.1371/journal.pone.0247908

**Published:** 2021-06-24

**Authors:** Shuang Gong, Zhen Wang, Lei Zhou

**Affiliations:** 1 School of Energy Science and Engineering, Henan Polytechnic University, Jiaozuo, China; 2 Henan Key Laboratory for Green and Efficient Mining & Comprehensive Utilization of Mineral Resources, Jiaozuo, China; 3 Collaborative Innovation Center of Coal Work Safety, Jiaozuo, Henan Province, China; Tianjin University, CHINA

## Abstract

To investigate the influence of bedding structure and different loading rates on the dynamic fracture characteristics and energy dissipation of Datong coal, a split Hopkinson bar was used to obtain the fracture characteristics of coal samples with different bedding angles. The process of crack initiation and propagation in Datong coal was recorded by the high-speed camera. The formula for the model I fracture toughness of the transversely isotropic material is obtained on the basis of the finite element method (FEM) together with the J-integral. By comparing the incident energy, absorbed energy, fracture energy and residual kinetic energy of Datong coal samples under various impact speeds, the energy dissipation characteristics during the dynamic fracture process of coal considering the bedding structure is acquired. The experimental results indicate that the fracture pattern of notched semi-circular bending (NSCB) Datong coal is tensile failure. After splitting into two parts, the coal sample rotates approximately uniformly around the contact point between the sample and the incident rod. The dynamic fracture toughness is 3.52~8.64 times of the quasi-static fracture toughness for Datong coal. Dynamic fracture toughness increases with increasing impact velocity, and the effect of bedding angle on fracture toughness then decreases. In addition, the residual kinetic energy of coal samples with the same bedding angle increases with the increase of impact speed. The energy utilization rate decreases continuously, and the overall dispersion of statistical data decreases gradually. In rock fragmentation engineering, the optimum loading condition is low-speed loading regardless of energy utilization efficiency or fracture toughness. These conclusions may have significant implications for the optimization of hydraulic fracturing process in coal mass and the further understanding of crack propagation mechanisms in coalbed methane extraction (CME). The anisotropic effect of coal should be fully considered in both these cases.

## 1. Introduction

The dynamic fracture toughness of coal refers to the ability of resisting brittle fracture of coal under high strain rate. Accurate acquisition of the dynamic fracture toughness is of great significance for coal and rock stability control, roadway support and rock burst prevention in mining engineering [1, 2]. Up to now, scholars have carried out a lot of dynamic fracture toughness testing research on rock materials using a split Hopkinson bar, including granite [3–7], marble [8–14], gabbro [8–10], limestone [15], asphalt mixture [16], sandstone [17–20], shale [19], concrete [20], ceramics [21] and glass [22]. However, there are few reports on the fracture characteristics of coal under dynamic loading.

In addition, according to the law of thermodynamics, the transformation of energy is the essential characteristic of the physical reaction of matter [23]. It is of great significance to study the energy dissipation law in the process of coal-rock fracture under impact load and the relationship between energy dissipation and coal-rock fracture instability. This is not only conducive to exploring the mechanism of dynamic disasters such as coal bump, coal and gas outburst and rock burst, but also can effectively improve the energy utilization efficiency of roadway excavation, borehole blasting and cutting from the perspective of energy utilization. In recent years, scholars have done a lot of research on the energy dissipation characteristic of rock fracture process, hoping to describe the deformation and failure behavior of rock from the energy point of view. The crack branching behaviors of gabbro and marble under different loading rates were analyzed by scanning electron microscopy. Moreover, the energy dissipation characteristics of the dynamic fracture process of short bar rock samples were measured by a split Hopkinson bar and high-speed camera [9]. Gao W X et al. carried out a plane impact test of sandstone using a single stage light gas gun, and calculated the damage energy dissipation density of sandstone under different impact loads [24]. Xu J Y et al. tested the energy dissipation characteristics of marble at different high temperatures by using a split Hopkinson pressure bar with high temperature device, and analyzed the relationship between fractal dimension of impact fragmentation and energy dissipation [25]. Huang D et al. carried out uniaxial compression tests of coarse-grained marble under nine different strain rates in the range of static loading rates. The effects of strain rates on stress-strain curves, failure modes, strength, elastic modulus, deformation modulus, energy consumption and release of strain were analyzed [26]. Chen K et al. investigated the evolution of multiphase microstructure and impact fracture behavior of medium carbon high silicon high strength steel subjected to the austempering treatment at 240, 360, and 400°C. The results show that the average carbon concentration in the matrix decreased with the temperature increase, while the impact toughness initially increased and then dropped with temperature [27]. Moreover, the fracture behaviors of aluminum, concrete, PMMA, silicon and other non-rock materials at quasi-static or dynamic rates has been extensively studied by numerical simulation and fracture toughness tests [28–32]. In summary, isotropic rock materials and other non-rock materials have been mostly used in previous studies, but there is little research on anisotropic coal-rock materials with bedding structure. The mechanical properties of coal show obvious anisotropy due to coal bedding, which is of great significance to the stability analysis of coal mass and roadway design. Therefore, it is necessary to test dynamic fracture characteristics and study the influence of coal bedding on its energy dissipation law.

The NSCB method and a split Hopkinson bar were used to test the dynamic fracture toughness of coal samples. The fracture modes and crack propagation behaviors of coal samples with different bedding angles under dynamic impact were fully analyzed. The formula for the model I fracture toughness of the transversely isotropic material is obtained on the basis of the finite element method (FEM) together with the J-integral. The fracture characteristics of coal samples with different bedding angles under different loading rates were tested and analyzed, and the rate response characteristics of dynamic fracture toughness of coal samples with different bedding angles were discussed. By calculating the incident energy, absorption energy, fracture energy and residual kinetic energy of coal samples under impact load, the energy dissipation law of dynamic fracture process of coal samples with different bedding angles under impact load was obtained.

## 2. Dynamic fracture test of coal

### 2.1. Specimen preparation

Coal samples were taken from No. 11 coal seam of Xinzhouyao Mine in Datong city. All samples were cut and processed from a complete coal block. Firstly, cylindrical cores with diameter of 50mm were drilled on parallel coal bedding surface, and then disc samples with thickness of 25mm were cut. Subsequently, each disc sample was cut into two half-disc samples along the diameter, and a 1 mm wide straight cutting groove was machined in the middle of the half-disc sample, and a diamond wire saw with a thickness of 0.1 mm was used to make the cutting tip. A total of 135 coal samples were prepared.

Before the dynamic fracture experiment, the conventional physical and mechanical properties of coal samples were tested according to the method recommended by the International Society of Rock Mechanics (ISRM), and the results are shown in Table 1. The quasi-static mechanical properties of the coal specimens were measured by an MTS815 hydraulic servo-controlled testing system with all measurements made at room temperature. The capacity of the load frame is 100 KN, and displacement control of 1.0 mm/min was used for the uniaxial compressive tests and 0.2 mm/min for the Brazilian split tensile tests. Industrial analysis of coal petrology shows that the moisture content of Datong coal is 2.92%, ash content is 25.34%, the volatile matter is 7.59%, and fixed carbon content is 64.15%. Based on the determination of the porosity by mercury injection, the porosity of coal is 27%.

**Table 1:** Summary of the physical and mechanical properties of Datong coal and the SHPB-bar.

Material	Density (kg/m^3^)	Cohesion (MPa)	Friction angle (°)	Young’s modulus (GPa)	Poisson’s ratio	Tensile strength (MPa)	Uniaxial compressive strength (MPa)
Coal	1301.06_5_±35.25	7.85_5_±0.91	32.64_5_±2.50	2.38_5_±0.22	0.38_5_±0.05	1.75_5_±0.03	27.64_5_±3.60
35CrMn Steel	7800	/	/	200	0.28	/	/

Note: The data in the table are expressed in the form of "Average value _Number of samples_ ± Standard deviation".

**Fig 1:**
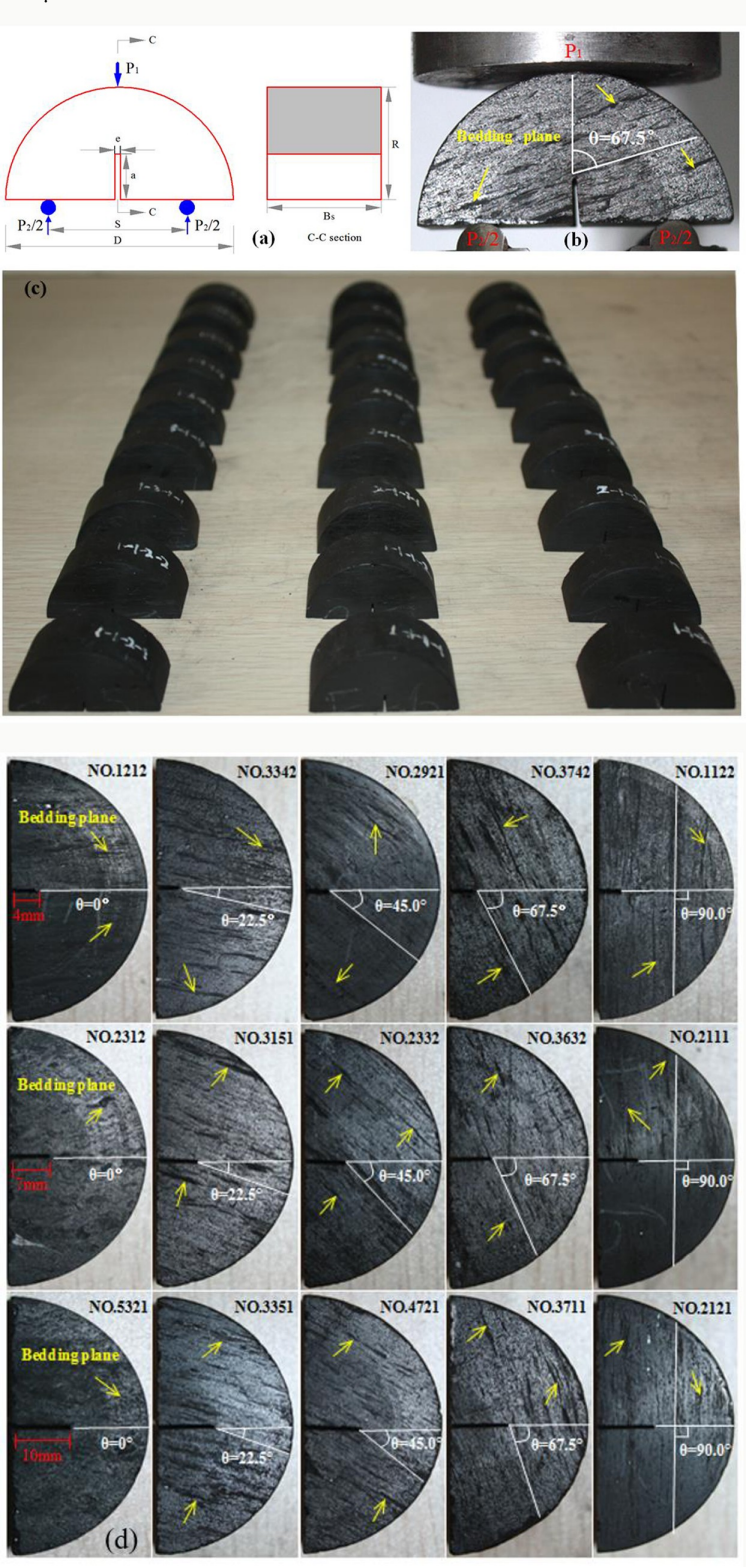
Schematic diagram of coal sample size and bedding angle. (a) Geometric dimension of coal sample. (b) Bedding angle. (c) Schematic diagram of some prepared samples. (d) Grouping according to different groove depths and bedding angles.

The geometric size of NSCB coal sample is shown in Fig 1(A), the width of groove *e* is 1 mm, the depth of groove *a* is designed into three groups (4mm, 7mm and 10mm), the distance between two supports is 30mm, the diameter of coal sample *D* is 50mm, and the thickness *B* is 25mm. Fig 1(B) is a typical bedding angle diagram of coal sample No.3731. According to the angle between bedding plane and impact direction, the coal samples are divided into five groups, which are 0°, 22.5°, 45°, 67.5° and 90°, respectively (see Fig 1(C) and 1(D)).

### 2.2. Experimental system

**Fig 2:**
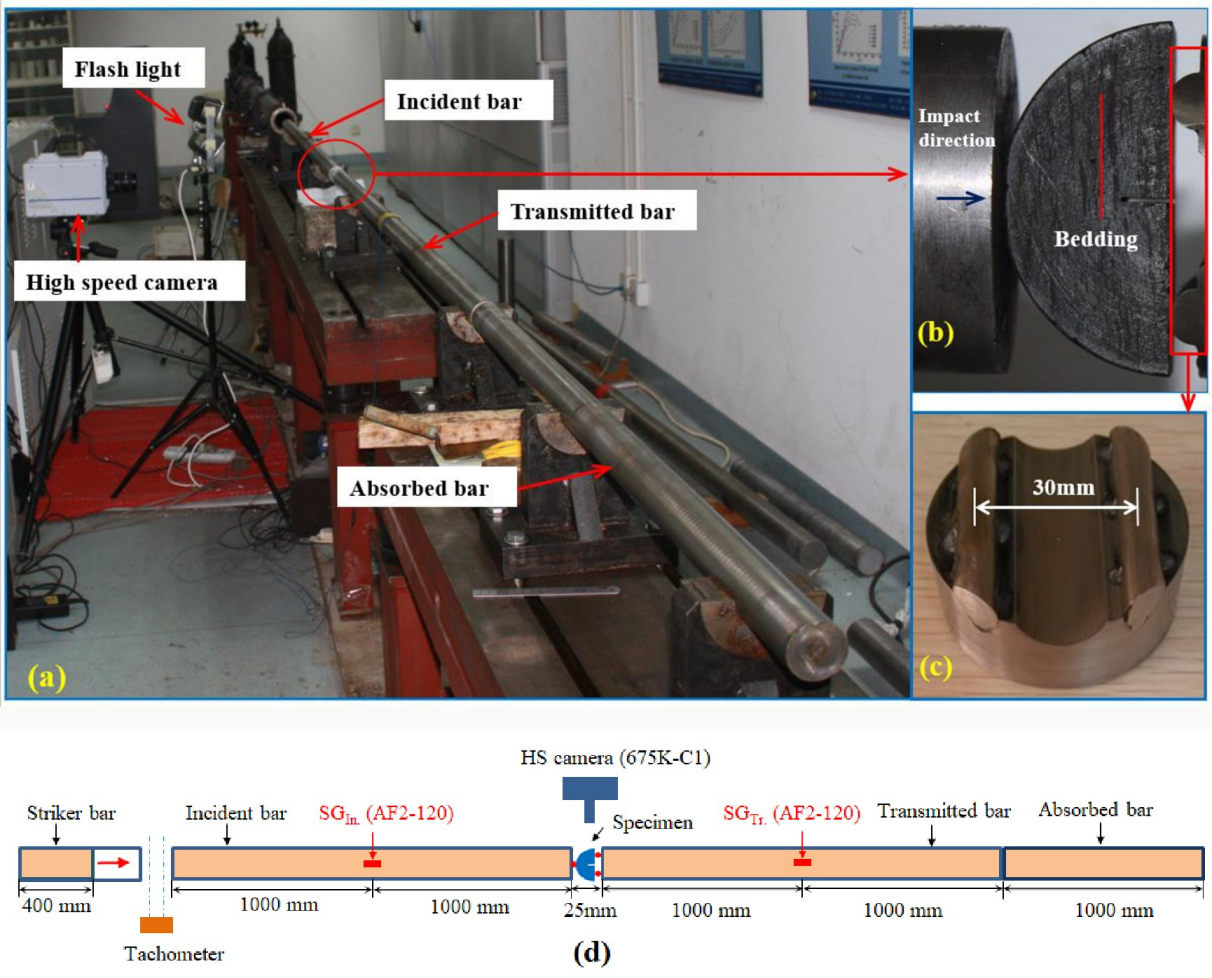
The SHPB loading apparatus. (a) Photograph of SHPB. (b) Impact direction and bedding angle. (c) Dimension of loading support. (d) Schematic diagram of SHPB.

The loading device for dynamic fracture toughness test of coal is a split Hopkinson pressure bar (SHPB) loading system. The SHPB dynamic rock mechanics test system is shown in Fig 2. The Hopkinson rod is made of 35CrMn Steel with a density of 7800 kg/m^3^, elastic modulus of 200GPa and Poisson’s ratio of 0.28 (Table 1). The length and diameter of input and output rods of SHPB loading system are 2m and 0.05m respectively. A strain gauge is attached to the middle of the input rod and the output rod to record the deformation of the rod.

In addition, the high-speed camera (Photron Fastcam SA1 with a spatial resolution of 256×192 pixels and a frame rate of 80,000 frames/s) was used to record the fracture process of NSCB coal sample under impact load. The trigger mode of high-speed camera is photoelectric post-trigger. The launching pressure of SHPB loading device is set to 0.50 MPa, 0.52 MPa and 0.54 MPa, so that the punch can produce different loading speeds. The surface brightness of the specimen was increased by flashing light to make the high-speed camera record the crack expansion process of the coal sample more clearly.

## 3. Results and discussion

### 3.1. Failure modes and characteristics of crack propagation

**Fig 3:**
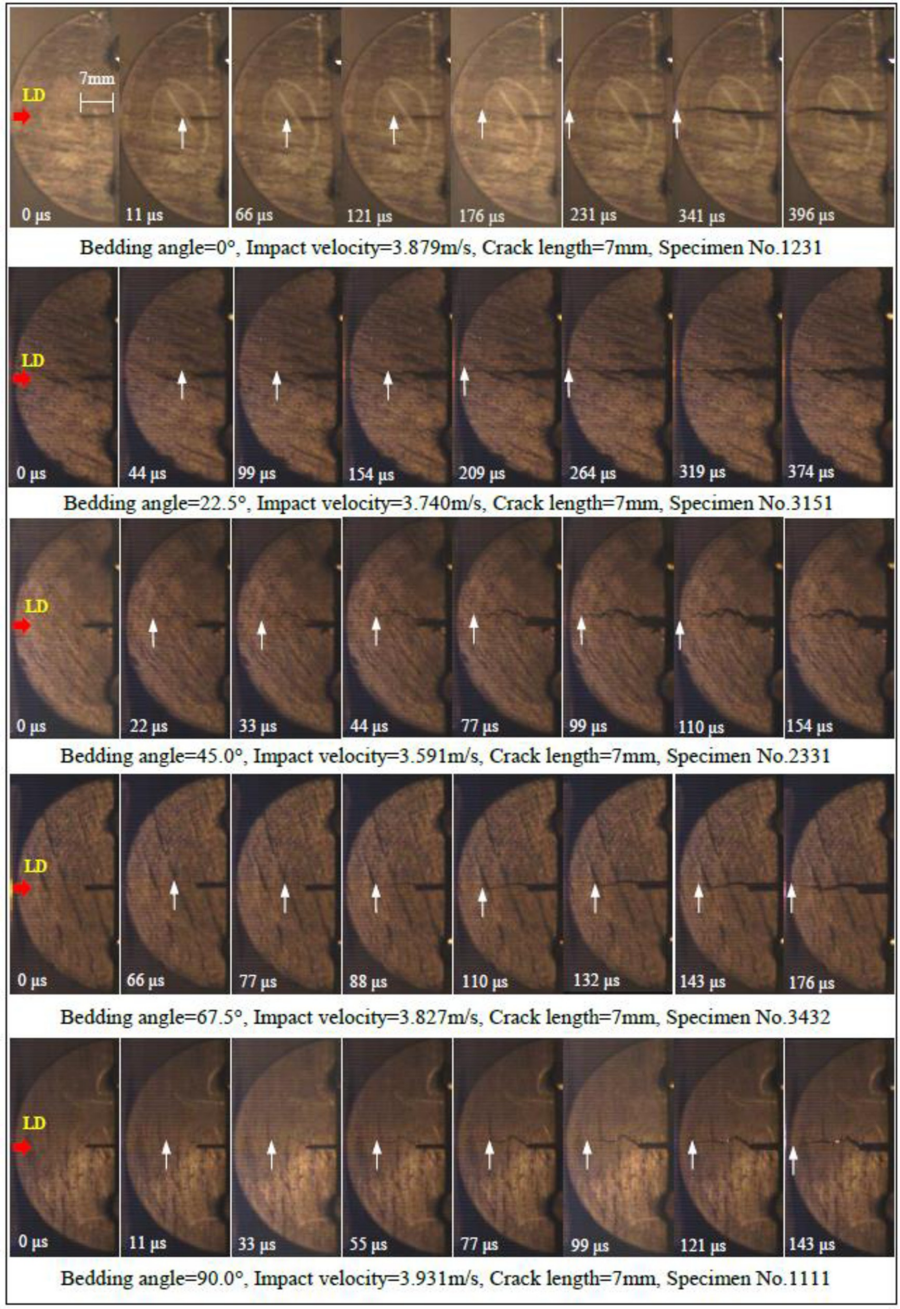
Dynamic fracture initiation and propagation process of coal with various bedding angles. (LD-loading direction, and the observable moving crack-tip is indicated by arrow in the images).

Fig 3 shows the dynamic fracture process of coal samples with different bedding angles under impact load. By analyzing the dynamic fracture process of 129 coal samples, it is found that the cracks mainly propagate along the plane of notch and impact direction, and the failure characteristics of coal samples are tensile failure. For instance, the crack propagation path of coal samples with 45° bedding angle is more complex than that of other samples. The crack first starts from the tip of the notch, then propagates along the weak bedding plane, and finally stops at the contact point between the incident bar and the sample. It should be noted that the crack propagation path is not straight due to the influence of the bedding plane, but presents an irregular fractal like feature. The coal sample is gradually stripped and decomposed along the bedding plane.

In addition, the crack propagation velocity of coal samples with different bedding angles can be calculated according to the crack initiation and penetration time recorded by high-speed camera, combined with the geometric size and groove depth of the sample. The crack propagation velocity of five groups of coal samples with different bedding angles in Fig 3 are 52.79, 68.18, 163.64, 102.27 and 125.87m/s respectively, with an average value of 102.55m/s. The ratio of the maximum crack propagation velocity to Rayleigh wave velocity is 0.216. The experimental results show that the crack growth rate of Datong coal sample is lower than that of Zhaogezhuang coal sample (234.03~324.88m/s) and laurenian granite (268~355m/s), and is about 7.76~24.06% of that of Fangshan marble sample (680m/s). The difference may be caused by different testing lithology or methods. It can be seen that the coal sample with 45° bedding angle has the largest crack growth rate, and the coal sample with 0° bedding angle has the smallest. Therefore, the bedding plane has a significant effect on the crack growth morphology and propagation rate of coal samples.

**Fig 4:**
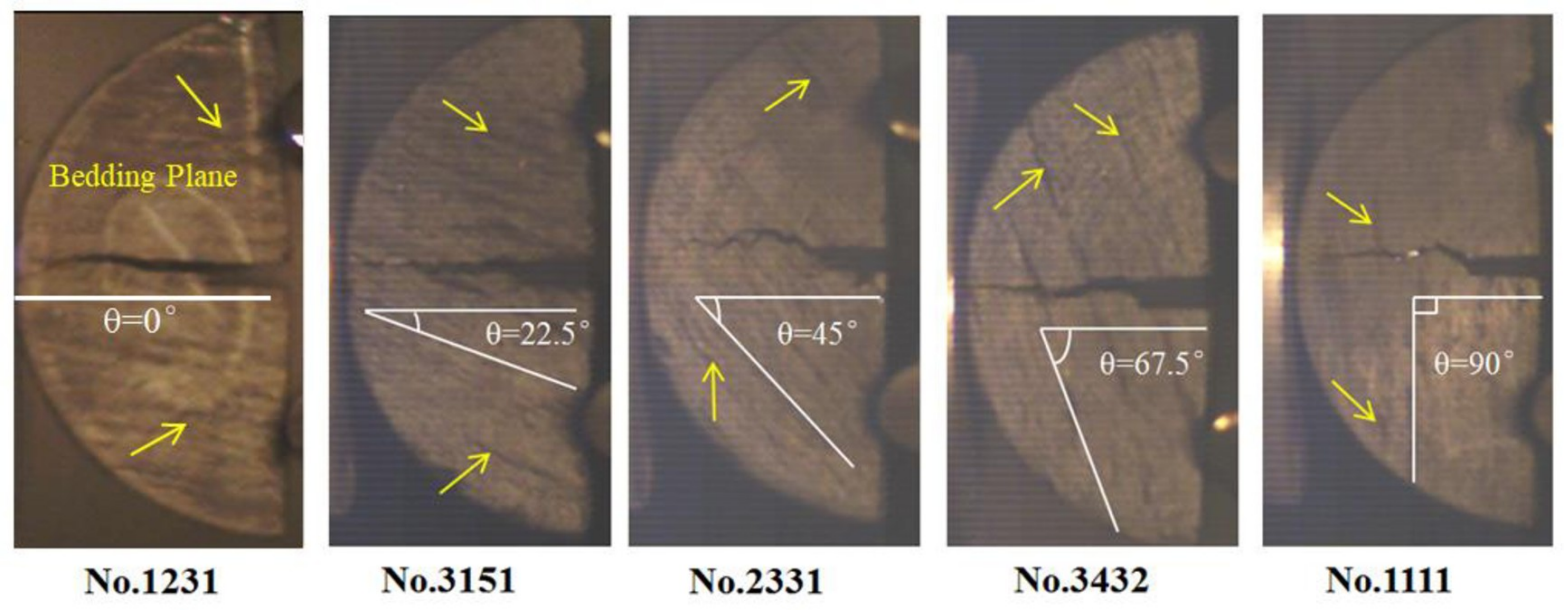
Fracture characteristics of coal samples with different bedding angles.

Fig 4 shows the final fracture morphology of coal samples. It can be seen that crack propagates approximately along the straight direction of the notch during fracture process of coal samples and the fracture mode is tensile failure. Moreover, the crack propagation path is irregular due to the influence of bedding angle of coal sample.

### 3.2. The dynamic force balance

According to the assumption of stress uniformity and the theory of three-wave method [33, 34], the dynamic stress-strain relationship in SHPB loading system is as follows:

$$\dot{\varepsilon }(t)=\frac{c}{l_{s}}(\varepsilon_{i}-\varepsilon_{r}-\varepsilon_{t})$$ (1)

$$\varepsilon (t)=\frac{c}{l_{s}}\int_{0}^{t}(\varepsilon_{i}-\varepsilon_{r}-\varepsilon_{t})dt$$ (2)

$$\sigma (t)=\frac{A}{2A_{s}}E(\varepsilon_{i}+\varepsilon_{r}+\varepsilon_{t})$$ (3)

In addition, based on the one-dimensional stress wave theory and the elastic deformation of the bars, the forces on the front and rear interfaces of the specimen can be expressed as:

$$P_{1}(t)=AE(\varepsilon_{i}+\varepsilon_{r})$$ (4)

$$P_{2}(t)=AE\varepsilon_{t}$$ (5)

where *ε*(*t*) is the strain measured by strain gauge on the bar, $\dot{\varepsilon }(t)$ is the strain rate, *σ*(*t*) is the dynamic stress history, *E* is the elastic modulus of the bar, *c* is the elastic wave velocity, *A* is the cross-sectional area of the bar, *A*_*s*_ is the initial cross-sectional area of the sample, *l*_*s*_ is the initial length of the sample. *ε*_*i*_, *ε*_*r*_ and *ε*_*t*_ are the incident, reflected, and transmitted strain.

**Fig 5:**
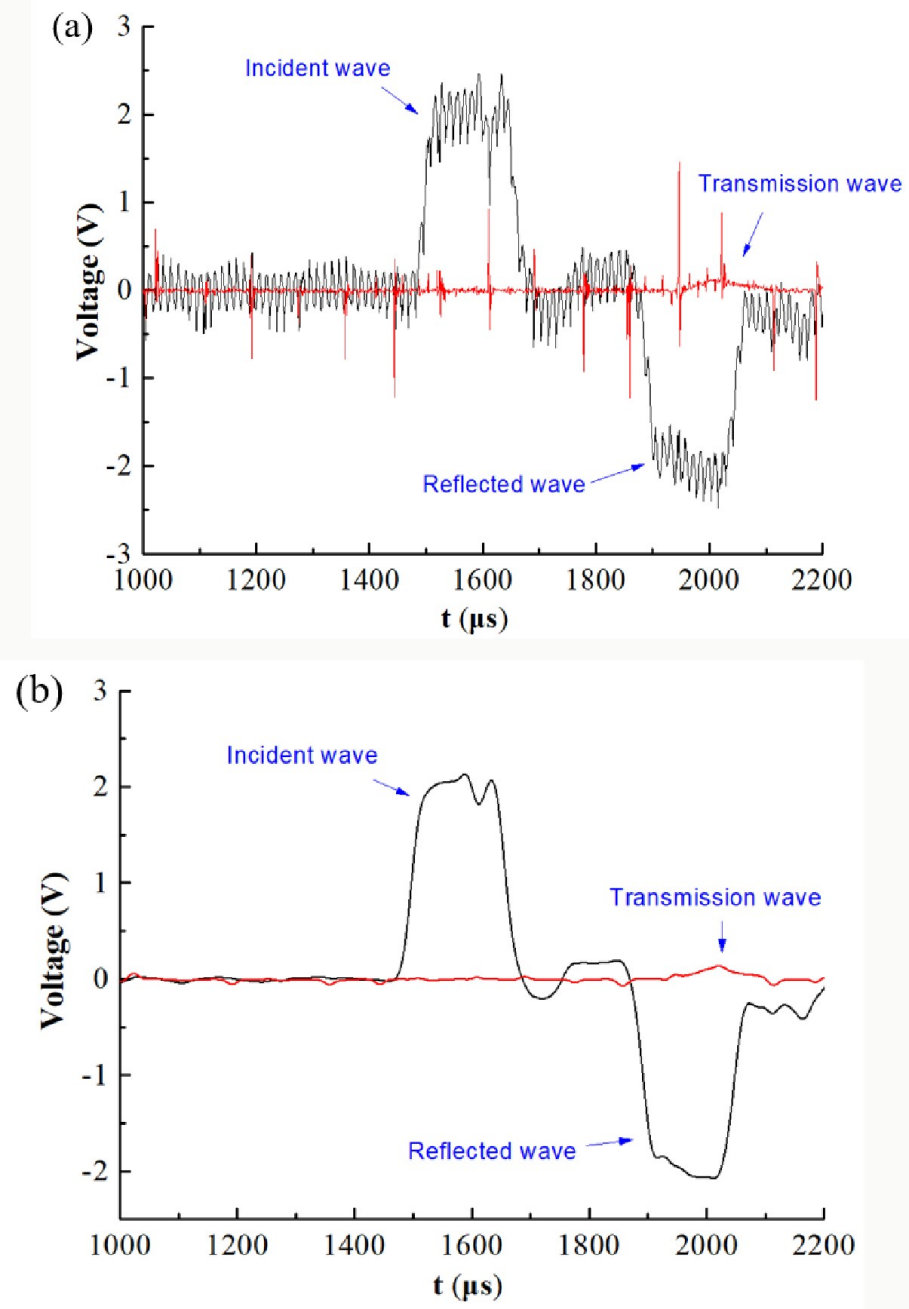
Curve of voltage change with time recorded by oscilloscope. (a) Voltage fluctuation curve with time before filtering. (b) Voltage fluctuation curve with time after filtering.

**Fig 6:**
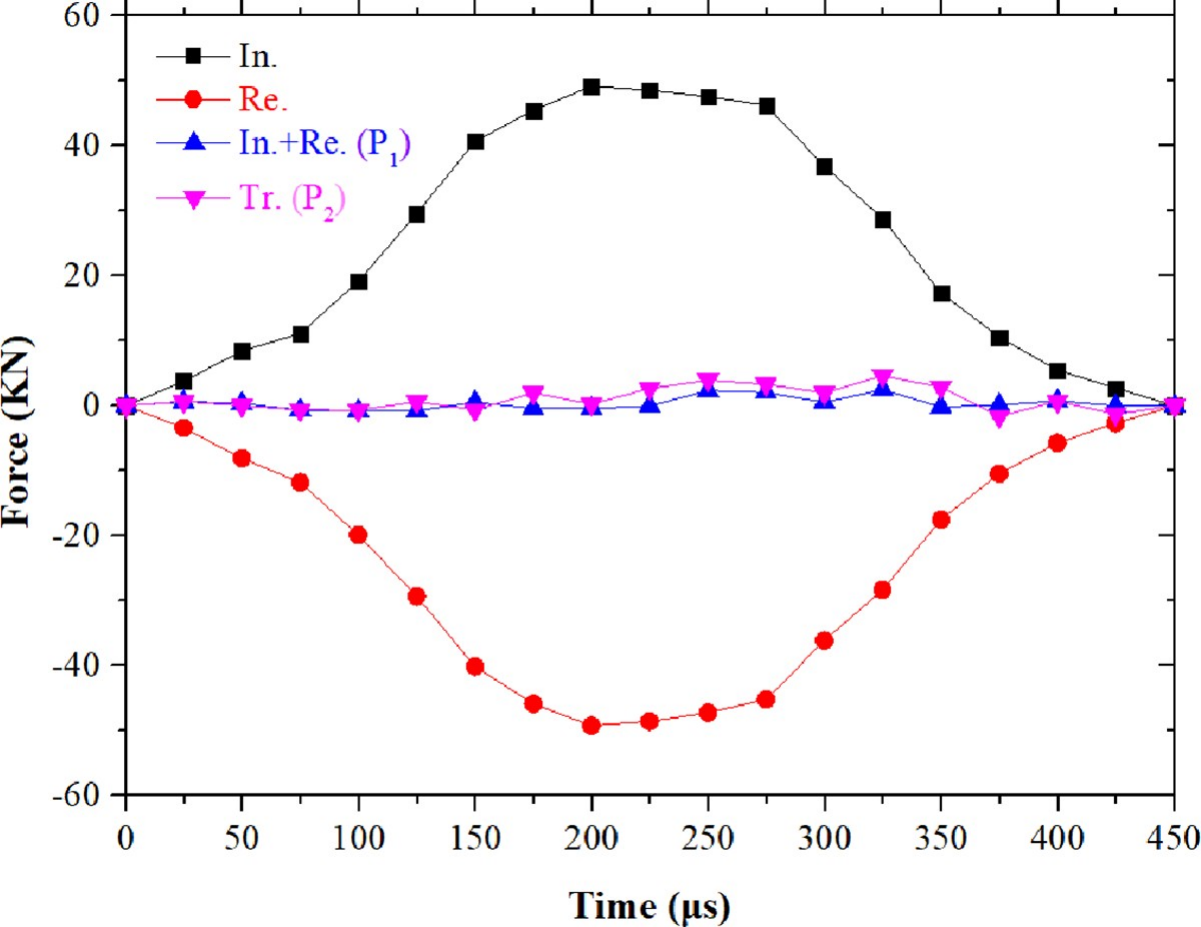
Dynamic force balance check for typical dynamic fracture toughness tests on the Datong coal (the symbols “In.” “Re.”, and “Tr.” represent the forces on the incident, reflected and transmitted bars, respectively).

The dynamic mechanical parameters of the coal specimen are obtained by calculating the voltage signal collected by the strain gauge on the SHPB system. The voltage signal data collected by strain gauge is output in the form of voltage-time curve. Fig 5(A) is the original waveform curve, and Fig 5(B) is the waveform curve processed by the filtering program. Before the impact test, a small amount of grease was applied to the contact part of the specimen and the loading bar as a lubricant to prevent the friction effect from interfering with the test results. In addition, a brass plate was pasted at the end of the incident bar as a waveform shaper to extend the loading wave and reduce the waveform oscillation, and this method can better obtain the ideal sinusoidal loading waveform. Fig 6 shows the dynamic loading history of two interfaces of a typical NSCB coal specimen. The dynamic force *P*_1_ and *P*_2_ are derived from Eqs (4) and (5). It can be seen that the condition of dynamic force equilibrium is fulfilled during the whole loading process. Before calculating the fracture toughness, the dynamic force equilibrium condition of each specimen was checked, and finally a total of 129 coal specimens passed the test and could be used for the next statistical analysis.

### 3.3. Calculation of dynamic fracture toughness

According to the test method of mode I fracture toughness for NSCB specimens recommended by the International Society of Rock Mechanics (ISRM), the calculation formula of fracture toughness *K*_*Id*_ is as follows [35]:

$$K_{Id}=\frac{P_{\max}S}{BR^{3/2}}Y(\alpha_{a},\alpha_{s})$$ (6)

where *P*_1_(*t*) and *P*_2_(*t*) are the loading forces at both ends of the specimen, *P*_max_ is the maximum value of *P*_1_(*t*) or *P*_2_(*t*) curve with time. *Y*(*α*_*a*_,*α*_*s*_) is the dimensionless stress intensity factor, which is a function of the geometry parameters *α*_*a*_ and *α*_*s*_, where *α*_*a*_ = *a*/*R*, *α*_*s*_ = *S*/2*R*.

**Fig 7:**
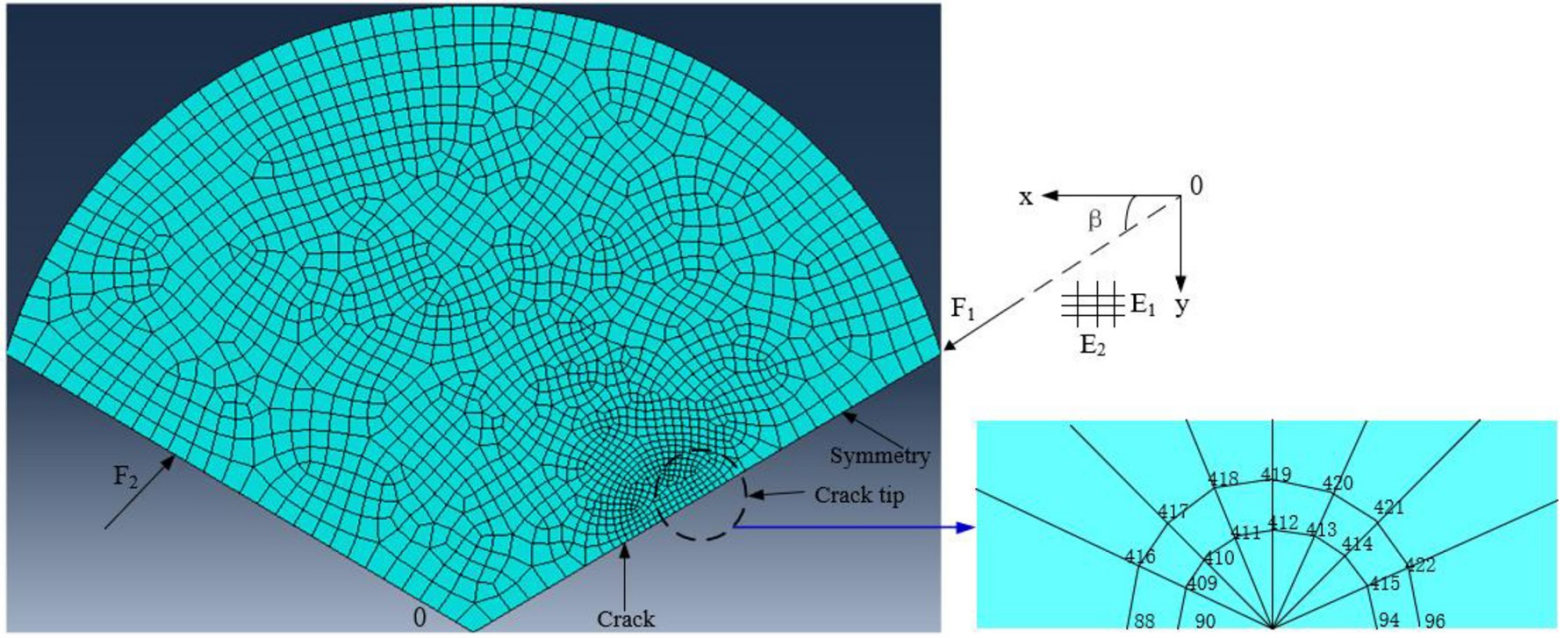
Modeling and meshing scheme for NSCB specimen by using FEM method.

It is noted that the above equations are only applicable to the calculation of isotropic elastic materials. However, for a transversely isotropic material such as coal, a new formula to calculate *Y*(*α*_*a*_,*α*_*s*_) needs to be derived to take into account its anisotropic characteristics [36–38]. In the present study, J-integral method was employed in a commercial FEM software (ANSYS 14.0) to obtain the *Y*(*α*_*a*_,*α*_*s*_) considering anisotropy. Fig 7 shows the modeling and meshing scheme for NSCB specimen. Due to the symmetry of the specimen geometry, only one half of the specimen needs to be built as a numerical model. The eight-node quadrilateral element PLAIN183 was adopted and the linear elastic orthogonal isotropic material model was used. The directions parallel to and perpendicular to the bedding plane are defined as the first and second principal directions of the material, respectively. The finite element model consists of 2252 elements and 7024 nodes (Fig 7). According to Eq (6), *Y* can be calculated by the following formula:

$$Y(\alpha_{a},\alpha_{s})=\frac{K_{Id}BR^{3/2}}{P_{\max}S}$$ (7)

where *α*_*s*_ = 0.6 based on ISRM standard [35].

**Fig 8:**
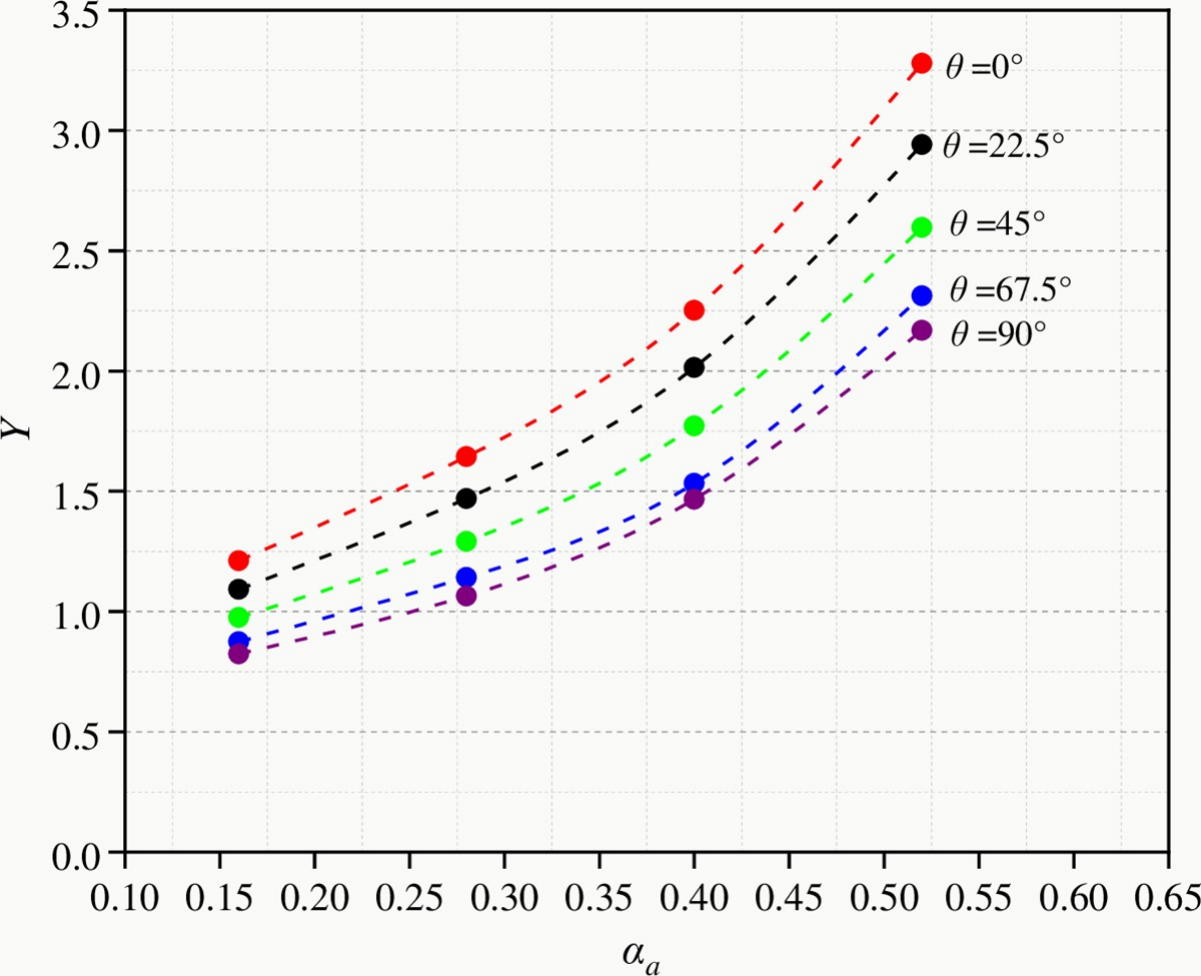
The fitting curves of *Y* varying with *α*_*a*_.

For five groups of coal samples, different loading angles *θ* between the loading direction and the principal direction of the material were applied to the FEM model. For a given notch length, the fracture toughness of crack tip and the peak load can be calculated. Then, *Y* can be calculated by Eq (7). The calculated data is shown in Table 2, and the relationship between *Y* and *α*_*a*_ is plotted in Fig 8.

**Table 2:** Dimensionless stress intensity factor with different notch lengths and loading angles calculated by FME.

Bedding angle (^o^)	Notch length (mm)	Dimensionless notch length (1)	Dimensionless stress intensity factor (1)
0.0	4	0.16	1.21
0.0	7	0.28	1.64
0.0	10	0.40	2.25
0.0	13	0.52	3.28
22.5	4	0.16	1.09
22.5	7	0.28	1.47
22.5	10	0.40	2.01
22.5	13	0.52	2.94
45.0	4	0.16	0.97
45.0	7	0.28	1.29
45.0	10	0.40	1.77
45.0	13	0.52	2.59
67.5	4	0.16	0.87
67.5	7	0.28	1.14
67.5	10	0.40	1.53
67.5	13	0.52	2.31
90.0	4	0.16	0.82
90.0	7	0.28	1.06
90.0	10	0.40	1.46
90.0	13	0.52	2.17

**Fig 9:**
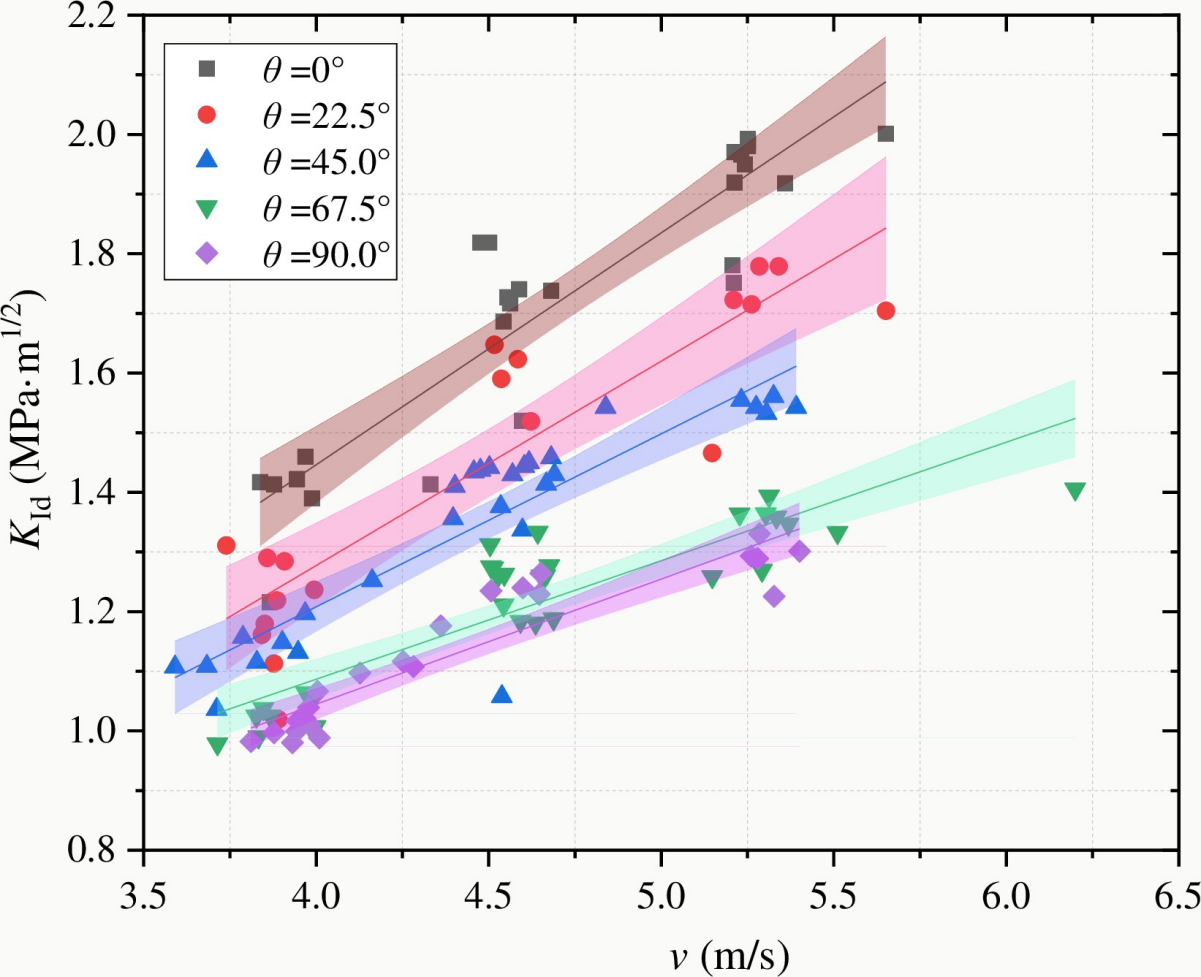
The relationship between dynamic fracture toughness and impact velocity of coal samples with different bedding angles (the different color areas correspond to the 95% confidence bands of the fracture toughness test values for each bedding angle grouping).

Fig 9 shows the relationship between dynamic fracture toughness and impact velocity of coal samples with different bedding angles (the data are from Tables 3–5). The different color areas correspond to the 95% confidence bands of the fracture toughness test values for each bedding angle grouping. It can be concluded that the dynamic fracture toughness decreases significantly with the increasing of the bedding angle, and the overall distribution is similar to a "step-like" one. However, in terms of the coal samples for each group of bedding angles, the fracture toughness increases continuously with the increase of impact velocity, showing a clear rate response characteristic [39–41]. The dynamic fracture toughness of coal sample is 0.85~2.2 MPa·m^-1/2^·s when the impact speed is 3.5~6.5m/s, which is 3.52~8.64 times of the quasi-static fracture toughness [33].

**Table 3:** Results of dynamic fracture toughness of coal samples (*α*_*a*_ = 0.16).

Specimen number	Bedding angle (^o^)	Loading velocity (m·s^-1^)	Dimensionless stress intensity factor (1)	Maximum load (KN)	Fracture toughness (MPa·m^1/2^)
1	0.0	3.866	1.211	3.303	1.215
2	0.0	3.969	1.211	3.967	1.459
3	0.0	3.988	1.211	3.777	1.389
4	0.0	4.543	1.211	4.583	1.685
5	0.0	4.562	1.211	4.666	1.716
6	0.0	4.681	1.211	4.725	1.738
7	0.0	5.207	1.211	4.840	1.780
8	0.0	5.210	1.211	4.759	1.750
9	0.0	5.212	1.211	5.218	1.919
10	0.0	5.251	1.211	5.415	1.991
11	22.5	3.843	1.092	3.500	1.160
12	22.5	3.851	1.092	3.555	1.179
13	22.5	3.887	1.092	3.674	1.218
14	22.5	3.891	1.092	3.073	1.019
15	22.5	3.995	1.092	3.726	1.236
16	22.5	4.623	1.092	4.579	1.519
17	22.5	5.148	1.092	4.419	1.465
18	22.5	5.210	1.092	5.193	1.722
19	45.0	3.712	0.975	3.500	1.036
20	45.0	3.902	0.975	3.877	1.148
21	45.0	3.948	0.975	3.821	1.131
22	45.0	4.397	0.975	4.579	1.355
23	45.0	4.402	0.975	4.763	1.410
24	45.0	4.457	0.975	4.844	1.434
25	45.0	4.535	0.975	4.648	1.376
26	45.0	4.617	0.975	4.896	1.449
27	45.0	5.326	0.975	5.271	1.560
28	67.5	3.971	0.874	4.006	1.063
29	67.5	3.987	0.874	3.780	1.003
30	67.5	3.998	0.874	3.792	1.006
31	67.5	4.504	0.874	4.941	1.311
32	67.5	4.636	0.874	4.447	1.180
33	67.5	4.642	0.874	5.021	1.332
34	67.5	4.653	0.874	4.745	1.259
35	67.5	4.664	0.874	4.745	1.259
36	67.5	5.313	0.874	5.250	1.393
37	67.5	5.334	0.874	5.116	1.358
38	90.0	3.967	0.823	4.082	1.021
39	90.0	3.978	0.823	4.154	1.039
40	90.0	4.004	0.823	4.262	1.066
41	90.0	4.599	0.823	4.958	1.240
42	90.0	4.651	0.823	5.055	1.264
43	90.0	5.285	0.823	5.320	1.330
44	90.0	5.401	0.823	5.202	1.301

**Table 4:** Results of dynamic fracture toughness of coal samples (*α*_*a*_ = 0.28).

Specimen number	Bedding angle (^o^)	Loading velocity (m·s^-1^)	Dimensionless stress intensity factor (1)	Maximum load (KN)	Fracture toughness (MPa·m^1/2^)
1	0.0	3.879	1.644	2.832	1.413
2	0.0	4.331	1.644	2.832	1.413
3	0.0	4.554	1.644	3.459	1.726
4	0.0	4.597	1.644	3.045	1.520
5	0.0	4.589	1.644	3.486	1.740
6	0.0	5.242	1.644	3.906	1.949
7	0.0	5.232	1.644	3.939	1.966
8	0.0	5.252	1.644	3.968	1.980
9	0.0	5.359	1.644	3.843	1.918
10	22.5	3.740	1.469	2.937	1.310
11	22.5	4.537	1.469	3.563	1.590
12	22.5	5.262	1.469	3.843	1.714
13	45.0	3.591	1.291	2.823	1.107
14	45.0	3.683	1.291	2.826	1.108
15	45.0	3.828	1.291	2.844	1.115
16	45.0	4.162	1.291	3.192	1.252
17	45.0	4.538	1.291	2.697	1.057
18	45.0	4.569	1.291	3.646	1.429
19	45.0	4.603	1.291	3.681	1.443
20	45.0	4.667	1.291	3.605	1.413
21	45.0	4.681	1.291	3.718	1.458
22	45.0	4.691	1.291	3.646	1.429
23	45.0	4.839	1.291	3.934	1.542
24	45.0	5.275	1.291	3.934	1.542
25	67.5	3.827	1.141	2.956	1.024
26	67.5	3.833	1.141	2.855	0.989
27	67.5	3.847	1.141	2.992	1.037
28	67.5	3.987	1.141	3.025	1.048
29	67.5	4.513	1.141	3.673	1.273
30	67.5	4.527	1.141	3.635	1.260
31	67.5	4.543	1.141	3.493	1.210
32	67.5	4.676	1.141	3.683	1.276
33	67.5	4.688	1.141	3.427	1.188
34	67.5	5.227	1.141	3.934	1.363
35	67.5	5.303	1.141	3.935	1.363
36	67.5	6.200	1.141	4.055	1.405
37	90.0	3.931	1.064	3.034	0.980
38	90.0	3.943	1.064	3.092	0.999
39	90.0	3.982	1.064	3.129	1.010
40	90.0	4.009	1.064	3.058	0.988
41	90.0	4.127	1.064	3.395	1.097
42	90.0	4.361	1.064	3.639	1.175
43	90.0	4.507	1.064	3.821	1.234
44	90.0	5.279	1.064	3.990	1.289

**Table 5:** Results of dynamic fracture toughness of coal samples (*α*_*a*_ = 0.40).

Specimen number	Bedding angle (^o^)	Loading velocity (m·s^-1^)	Dimensionless stress intensity factor (1)	Maximum load (KN)	Fracture toughness (MPa·m^1/2^)
1	0.0	3.838	2.252	2.072	1.416
2	0.0	3.945	2.252	2.080	1.422
3	0.0	4.502	2.252	2.659	1.818
4	0.0	4.476	2.252	2.659	1.818
5	0.0	5.212	2.252	2.881	1.970
6	0.0	5.232	2.252	2.881	1.970
7	0.0	5.252	2.252	2.908	1.988
8	0.0	5.651	2.252	2.926	2.001
9	22.5	3.859	2.014	2.109	1.289
10	22.5	3.878	2.014	1.819	1.112
11	22.5	3.909	2.014	2.100	1.284
12	22.5	4.517	2.014	2.694	1.647
13	22.5	4.585	2.014	2.654	1.623
14	22.5	5.285	2.014	2.909	1.778
15	22.5	5.341	2.014	2.909	1.778
16	22.5	5.652	2.014	2.787	1.704
17	45.0	3.788	1.771	2.151	1.156
18	45.0	3.968	1.771	2.225	1.196
19	45.0	4.476	1.771	2.672	1.437
20	45.0	4.502	1.771	2.680	1.441
21	45.0	4.598	1.771	2.486	1.336
22	45.0	5.232	1.771	2.891	1.554
23	45.0	5.305	1.771	2.849	1.532
24	45.0	5.391	1.771	2.868	1.542
25	67.5	3.714	1.532	2.102	0.978
26	67.5	3.867	1.532	2.201	1.024
27	67.5	4.545	1.532	2.712	1.262
28	67.5	4.507	1.532	2.740	1.275
29	67.5	4.592	1.532	2.543	1.183
30	67.5	5.148	1.532	2.704	1.258
31	67.5	5.292	1.532	2.727	1.269
32	67.5	5.369	1.532	2.893	1.346
33	67.5	5.511	1.532	2.864	1.332
34	90.0	3.811	1.466	2.206	0.982
35	90.0	3.878	1.466	2.238	0.996
36	90.0	3.945	1.466	2.283	1.016
37	90.0	4.282	1.466	2.488	1.108
38	90.0	4.252	1.466	2.507	1.116
39	90.0	4.647	1.466	2.760	1.229
40	90.0	5.261	1.466	2.904	1.293
41	90.0	5.327	1.466	2.751	1.225

### 3.4. Energy partitioning in dynamic fracture process of coal

Previous studies have shown that the effective energy used for rock breakage and fragmentation in mining is far less than the actual input energy. To improve efficiency, it is essential to quantitatively evaluate the energy distribution during the dynamic fracture of coal. In the SHPB experiment, the energies of the incident wave *W*_*In*._, the reflected wave *W*_*Re*._, and the transmitted wave *W*_*Tr*._ are expressed respectively as [35]

WIn.=ABCBEB∫σIn.(t)2dt,WRe.=ABCBEB∫σRe.(t)2dt,WTr.=ABCBEB∫σTr.(t)2dt (8)

where *A*_*B*_ is the cross-sectional area of the bars, *C*_*B*_ is the longitudinal wave speed, and *E*_*B*_ is the Young’s modulus of the bars, and *σ* is the stress measured by strain gauges on the bars.

Lubricant is used at the interface between the rod and the specimen to reduce the frictional effects. Dai et al. [7] also indicated that the coefficient of friction of the NSCB method was about 0.02 in the well-lubricated SHPB test. Thus, the friction can be neglected and the total energy absorbed by the sample is [35]:

WB=WIn.−WRe.−WTr. (9)

**Fig 10:**
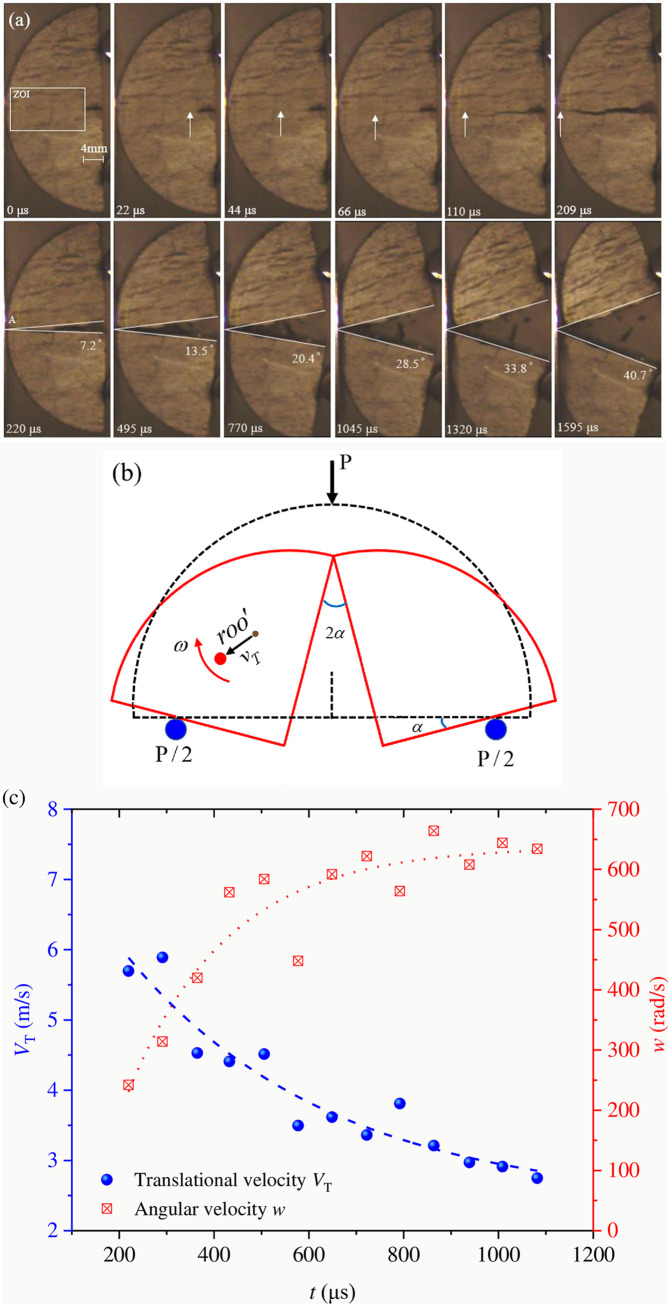
(a) Surface crack propagation and rotation process of coal sample. (b) Schematic diagram of the fracture process in coal sample (*O* is the center of mass, *roo*′ is the distance of translation movement). (c) Translational and rotational angular velocities of coal samples during fracture.

Fig 10(A) shows a high-speed photograph of the coal rock, and the result shows that the NSCB specimen splits into two almost equal pieces, and each flying piece has both rotational and translational motion. When *t* = 22μs, the crack initiate at the notch tip, and then begins to propagate. The front of the crack is shown by the arrow in the figure. When *t* = 110μs, the crack penetrates through the NSCB coal sample, and the sample splits into two halves. The destroyed coal sample continues to rotate around the contact point A between the sample and the incident bar, and its rotation angle in *t* = 220~1595μs is shown in the figure. It can be concluded that the angular velocity of coal sample rotating around A after failure is 336.2–513.8 rad/s, and the average angular velocity is 425 rad/s, which is approximately uniform rotation. The results are similar to those of NSCB granite samples tested in the past [40], but the rotational speed of coal sample is greater than that of granite (about 314 rad/s).

The translation velocity *V*_T_ can be calculated from the translation distance of the center of mass *r*_*oo*_′ and the corresponding time recorded by the high-speed camera image. The angular velocity *w* is calculated based on the rotation angle *α* and the corresponding time, as shown in Fig 10(B). The local coordinates of the center of mass and the rotation angle of the fragments can be quantified from the relative photographs at each time step. In the initial stage, *V*_T_ tends to decrease, but *w* increases with the time, as shown in Fig 10(C), which clearly indicates that *w* increases at the expense of *V*_T_ decrease. These two velocities eventually converge to a constant, which indicates that the overall kinetic energy is invariant. From the velocities corresponding to each time step of each fragment, the kinetic energy of rotation *T*_Rot._ and the translational energy *T*_Tra._ are obtained as follows:

$$T_{Rot.}=\frac{1}{2}Iw^{2}$$

$$T_{Tra.}=\frac{1}{2}mv_{T}^{2}$$ (10)

The overall kinetic energy *T* of the two fragments is

$$T=2(T_{Tra.}+T_{Rot.})=mv_{T}^{2}+Iw^{2}$$ (11)

where *m* is the mass of a single fragment, and $I=\frac{R^{2}}{36\pi^{2}}(9\pi^{2}+18\pi -128)m$ is the rotational inertia around the axis of rotation.

The energy absorbed by the coal sample (*W*_B_) consists of three main components: (1) kinetic energy (*T*) of the two fragments; (2) fracture and damage energy (*W*_G_) to produce fracture surfaces and bifurcation cracks, and to produce damage such as microscopic cracks in the coal; (3) energy consumed in other forms (*W*_O_), such as thermal energy.

Zhang et al. concluded that at low loading rates (less than 1.0×10^6^ MPa m^1/2^ s^-1^), the thermal energy (*W*_O_) is small and can be neglected in the energy calculations [9]. Therefore, the following equation can be derived

$$W_{B}=T+W_{G}$$ (12)

To analyze the effects of impact speed and bedding angle on incident energy, absorption energy, fracture energy and residual kinetic energy of coal samples, 54 energy dissipation data of coal samples were obtained. Table 6 shows the statistical distribution of characteristic parameters of energy dissipation in coal samples. The incident energy of coal sample is greatly affected by the impact speed. The average impact speed of coal sample with bedding angle of 0 degree is the largest, the incident energy is the largest and the variance is the highest, while the incident energy of coal sample with bedding angle of 22.5 degree is the smallest. The average impact speed of coal sample with bedding angle of 67.5 degree is the smallest, and the variance of incident energy is the lowest. Absorption energy, fracture energy and residual kinetic energy of coal sample with 0 degree bedding angle are the largest, while that of coal sample with 22.5 degree bedding angle is the smallest. Energy dissipation rate of coal sample with 67.5 degree bedding angle is the highest, and that of coal sample with 22.5 degree bedding angle is the smallest. This is due to the difference between the absorption energy and residual kinetic energy of coal samples. It can be seen that the absorption energy of coal samples with 22.5 degree bedding angle is the smallest in each group, resulting in smallest fracture energy and residual kinetic energy (Table 6). The energy dissipation rate is the ratio of fracture energy to absorbed energy (*W*_*G*_/*W*_*B*_). The fracture energy of coal sample with bedding angle of 22.5 degree is the smallest, but the kinetic energy of impact rod in each group is similar, which results in the lowest energy dissipation rate of coal sample with bedding angle of 22.5 degree.

**Table 6:** Energy dissipation statistics of coal samples.

Bedding angle (°)	Impact speed (m·s^-1^)	Incident energy (J)	Absorption energy (J)	Impact rod kinetic energy (J)	Fracture energy (J)	Residual kinetic energy (J)	Energy dissipation rate (%)
0.0	4.826_11_±0.54	12.870_11_±4.41	1.984_11_±0.48	72.14_11_±15.6	1.408_11_±0.44	0.576_11_±0.24	2.04_11_±0.69
22.5	4.324_11_±0.58	5.137_11_±2.32	0.936_11_±0.46	58.17_11_±16.1	0.748_11_±0.39	0.188_11_±0.09	1.33_11_±0.72
45.0	4.370_10_±0.59	6.741_10_±2.62	1.667_10_±0.65	59.44_10_±16.2	1.311_10_±0.55	0.356_10_±0.22	2.24_10_±0.91
67.5	4.114_6_±0.37	5.755_6_±0.85	1.521_6_±0.75	52.15_6_±9.5	1.202_6_±0.72	0.319_6_±0.11	2.29_6_±1.42
90.0	4.308_16_±0.47	5.468_16_±1.99	1.441_16_±0.66	57.47_16_±13.3	1.164_16_±0.61	0.277_16_±0.14	2.08_16_±1.19

Note: The data in the table are expressed in the form of "Average value _Number of samples_ ± Standard deviation".

**Fig 11:**
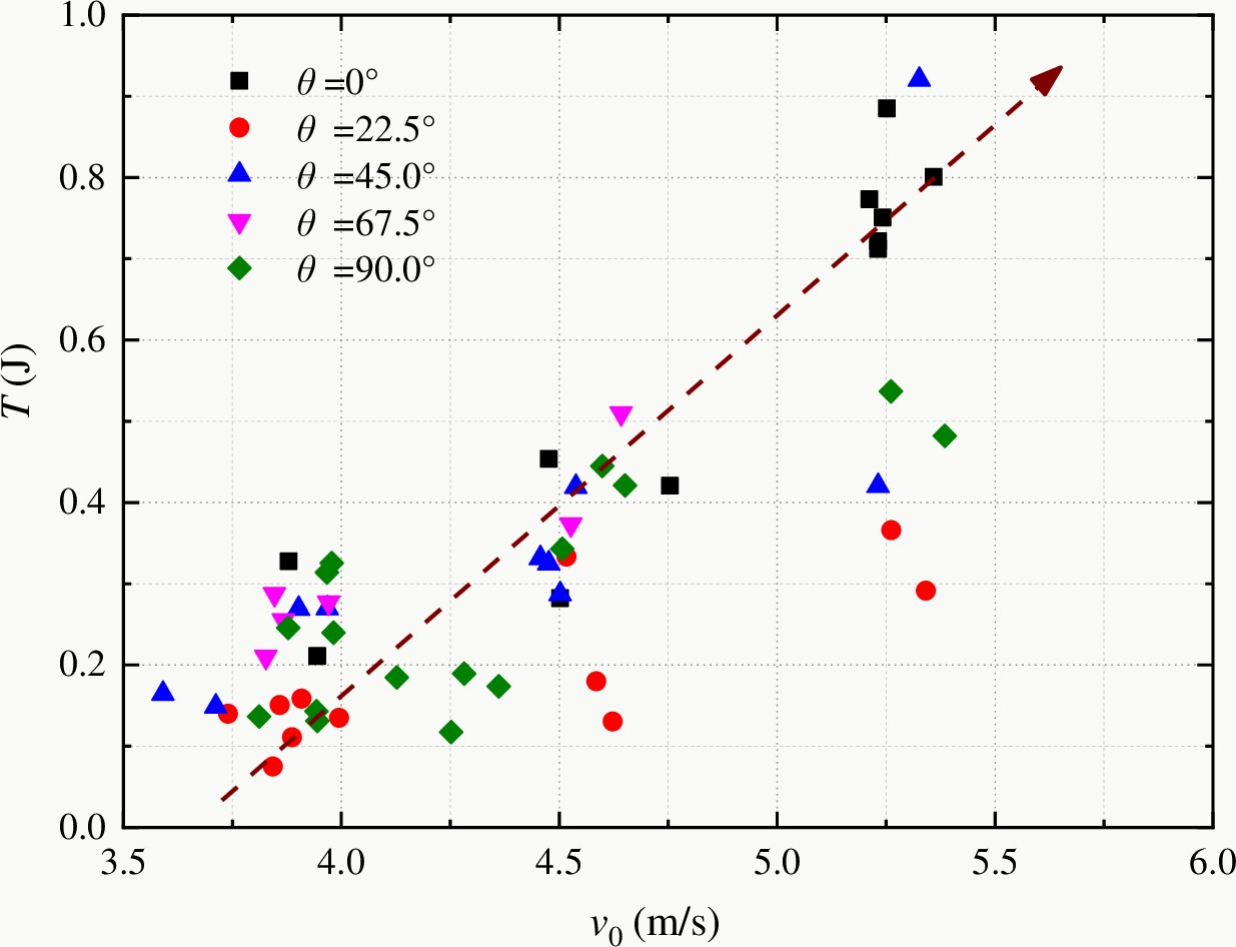
Change of kinetic energy with impact speed of the striker bar (the dashed arrow in the figure shows the overall change trend of kinetic energy with impact velocity).

Fig 11 shows the variation of residual kinetic energy with impact speed of coal samples with different bedding angles. When the impact speed is 3.5–5.5 m/s, residual kinetic energy of coal sample is in the range of 0.11–0.93 J. Residual kinetic energy of coal samples with the same bedding angle increases with the increase of impact speed. This conclusion is consistent with the results of residual kinetic energy measurements of gabbro and marble [9].

**Fig 12:**
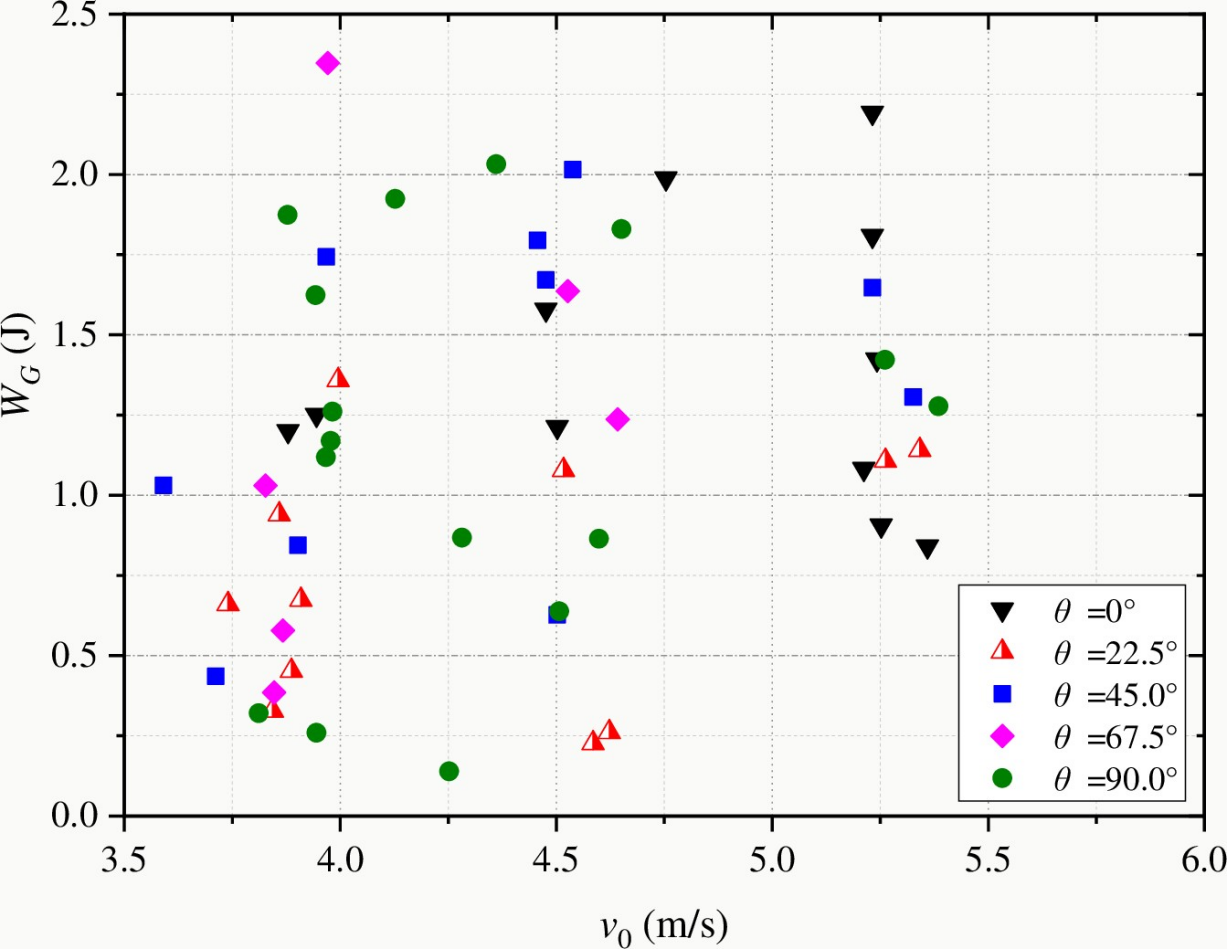
Change of fracture energy with impact speed of striker bar.

**Fig 13:**
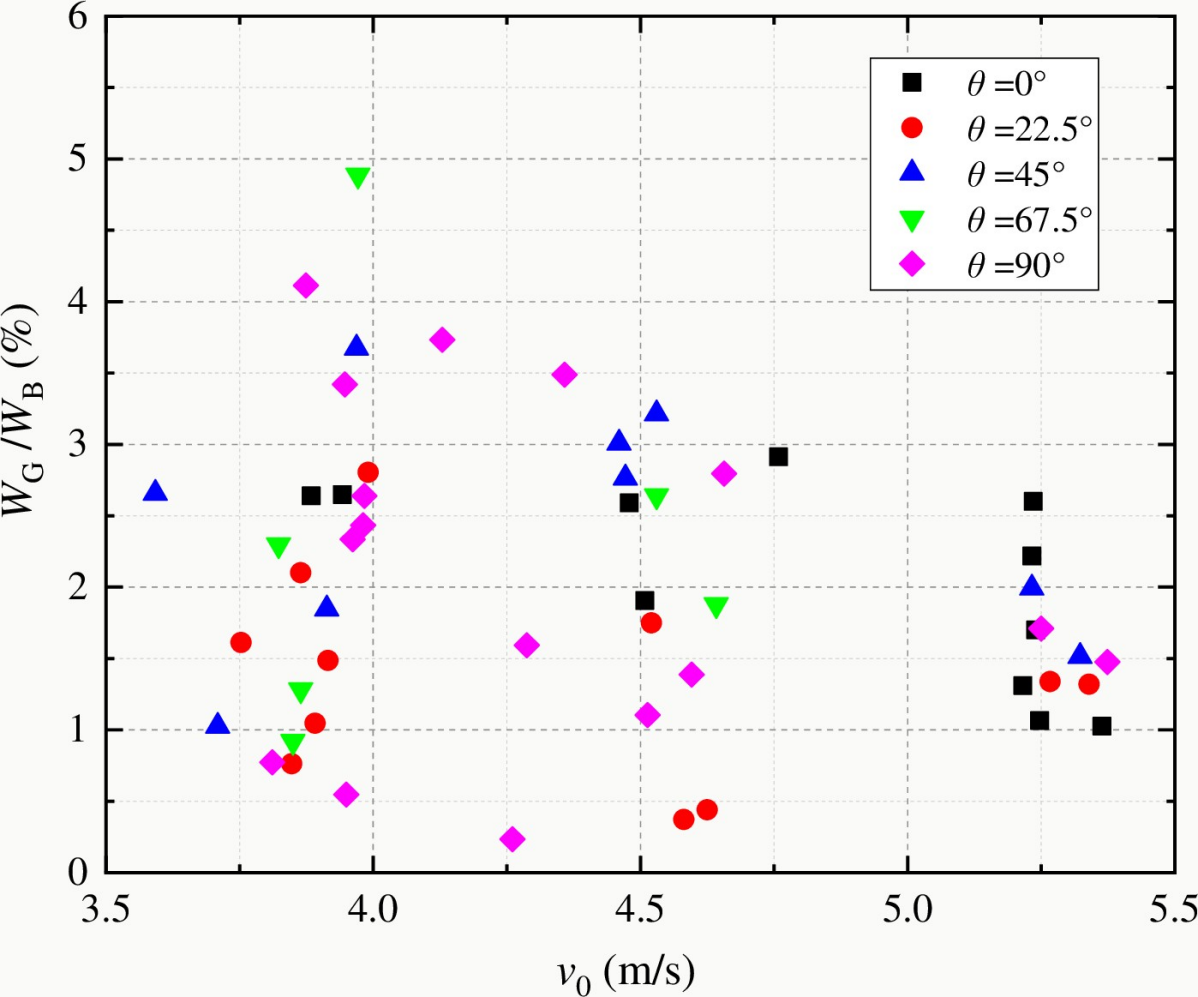
Change of energy utilization ratio with impact speed of striker bar.

Fig 12 shows the variation of fracture energy with impact speed of coal samples with different bedding angles. It can be seen that the test results are discrete due to the composition and structural characteristics of coal. This is because under the condition of high-speed impact load, more macro-bifurcation cracks and micro-damage cracks will be produced in the coal sample, resulting in more energy consumption in the fracture process [9]. Fig 13 shows the variation of energy dissipation rate (*W*_*G*_/*W*_*B*_) with impact speed during dynamic fracture of coal samples. With the increase of impact speed, the energy utilization rate decreases, and the overall dispersion of statistical data decreases. Therefore, from the point of view of energy utilization efficiency, the best condition for crushing coal and rock is low-speed loading, so the overall energy utilization efficiency is higher. On the other hand, from the point of view of fracture toughness, according to the rate response characteristics of fracture toughness, that is, fracture toughness increases with the increase of impact speed. Fractured coal and rock should also be under low-speed loading conditions, so that the fracture toughness of coal and rock is smaller and more fragile. To sum up, in the process of rock drilling, excavation or crushing, the best energy utilization method is low-speed loading.

## 4. Conclusion

During the dynamic fracture process of NSCB Datong coal samples, the cracks propagate approximately along the straight-line direction of the notch, mainly as tensile failure. Moreover, the crack propagation path is irregular due to the influence of bedding angle of coal sample. The rotation angle of Datong coal samples with various bedding angles increases linearly with time and exhibits uniform rotation characteristics.

Dynamic fracture toughness of Datong coal sample is 0.85~1.7MPa·m^-1/2^·s when the impact speed is 3.5~6.5m/s, which is 3.52~8.64 times of the quasi-static fracture toughness. With the increase of impact speed, dynamic fracture toughness increases, but the influence of bedding angle on fracture toughness decreases. Impact speed is the main factor affecting dynamic fracture toughness of Datong coal samples.

With the increase of impact speed, the residual kinetic energy of coal samples with the same bedding angle increases, and the dynamic fracture energy also increases. The energy utilization rate decreases continuously, and the overall dispersion of statistical data decreases gradually.

In rock fragmentation engineering, the optimum loading condition is low-speed loading regardless of energy utilization efficiency or fracture toughness. Because the energy utilization efficiency is higher, and the fracture toughness of rock is smaller and easier to break.

## Supporting information

S1 Table Summary of the physical and mechanical properties of Datong coal and the SHPB-bar. (DOCX)

S2 Table Dimensionless stress intensity factor with different notch lengths and loading angles calculated by FME. (DOCX)

S3 Table Results of dynamic fracture toughness of coal samples (*α*_*a*_ = 0.16). (DOCX)

S4 Table Results of dynamic fracture toughness of coal samples (*α*_*a*_ = 0.28). (DOCX)

S5 Table Results of dynamic fracture toughness of coal samples (*α*_*a*_ = 0.40). (DOCX)

S6 Table Energy dissipation statistics of coal samples. (DOCX)
