# Supplementary material for: Dynamic fracture mechanics and energy distribution rate response characteristics of coal containing bedding structure
Source: PLoS One. 2021 Jun 24;16(6):e0247908. doi: 10.1371/journal.pone.0247908 (PMC8224884; doi:10.1371/journal.pone.0247908)
Supplement: S1 Table — (DOCX) [file pone.0247908.s001.docx]

**Table 1** Summary of the physical and mechanical properties of Datong coal and the SHPB-bar.

| Material | Density  (kg/m^3^) | Cohesion  (MPa) | Friction angle  (°) | Young’s modulus  (GPa) | Poisson’s ratio | Tensile strength  (MPa) | Uniaxial compressive strength  (MPa) |
| --- | --- | --- | --- | --- | --- | --- | --- |
| Coal | 1301.06_5_±35.25 | 7.85_5_±0.91 | 32.64_5_±2.50 | 2.38_5_±0.22 | 0.38_5_±0.05 | 1.75_5_±0.03 | 27.64_5_±3.60 |
| 35CrMn Steel | 7800 | / | / | 200 | 0.28 | / | / |

*Note: The data in the table are expressed in the form of "**Average value* *_Number of samples_ ± Standard deviation".*
