# Supplementary material for: Dynamic fracture mechanics and energy distribution rate response characteristics of coal containing bedding structure
Source: PLoS One. 2021 Jun 24;16(6):e0247908. doi: 10.1371/journal.pone.0247908 (PMC8224884; doi:10.1371/journal.pone.0247908)
Supplement: S2 Table — (DOCX) [file pone.0247908.s002.docx]

**Table 2** Dimensionless stress intensity factor with different notch lengths and loading angles calculated by FME.

| Bedding angle  (^o^) | Notch length  (mm) | Dimensionless  notch length (1) | Dimensionless stress intensity factor (1) |
| --- | --- | --- | --- |
| 0.0 | 4 | 0.16 | 1.21 |
| 0.0 | 7 | 0.28 | 1.64 |
| 0.0 | 10 | 0.40 | 2.25 |
| 0.0 | 13 | 0.52 | 3.28 |
| 22.5 | 4 | 0.16 | 1.09 |
| 22.5 | 7 | 0.28 | 1.47 |
| 22.5 | 10 | 0.40 | 2.01 |
| 22.5 | 13 | 0.52 | 2.94 |
| 45.0 | 4 | 0.16 | 0.97 |
| 45.0 | 7 | 0.28 | 1.29 |
| 45.0 | 10 | 0.40 | 1.77 |
| 45.0 | 13 | 0.52 | 2.59 |
| 67.5 | 4 | 0.16 | 0.87 |
| 67.5 | 7 | 0.28 | 1.14 |
| 67.5 | 10 | 0.40 | 1.53 |
| 67.5 | 13 | 0.52 | 2.31 |
| 90.0 | 4 | 0.16 | 0.82 |
| 90.0 | 7 | 0.28 | 1.06 |
| 90.0 | 10 | 0.40 | 1.46 |
| 90.0 | 13 | 0.52 | 2.17 |
