# Supplementary material for: Dynamic fracture mechanics and energy distribution rate response characteristics of coal containing bedding structure
Source: PLoS One. 2021 Jun 24;16(6):e0247908. doi: 10.1371/journal.pone.0247908 (PMC8224884; doi:10.1371/journal.pone.0247908)
Supplement: S3 Table — (DOCX) [file pone.0247908.s003.docx]

Table 3 Results of dynamic fracture toughness of coal samples (*α_a_* = 0.16).

| Specimen number | Bedding angle  (^o^) | Loading velocity (m·s^-1^) | Dimensionless stress intensity factor (1) | Maximum load  (KN) | Fracture toughness (MPa·m^1/2^) |
| --- | --- | --- | --- | --- | --- |
| 1 | 0.0 | 3.866 | 1.211 | 3.303 | 1.215 |
| 2 | 0.0 | 3.969 | 1.211 | 3.967 | 1.459 |
| 3 | 0.0 | 3.988 | 1.211 | 3.777 | 1.389 |
| 4 | 0.0 | 4.543 | 1.211 | 4.583 | 1.685 |
| 5 | 0.0 | 4.562 | 1.211 | 4.666 | 1.716 |
| 6 | 0.0 | 4.681 | 1.211 | 4.725 | 1.738 |
| 7 | 0.0 | 5.207 | 1.211 | 4.840 | 1.780 |
| 8 | 0.0 | 5.210 | 1.211 | 4.759 | 1.750 |
| 9 | 0.0 | 5.212 | 1.211 | 5.218 | 1.919 |
| 10 | 0.0 | 5.251 | 1.211 | 5.415 | 1.991 |
| 11 | 22.5 | 3.843 | 1.092 | 3.500 | 1.160 |
| 12 | 22.5 | 3.851 | 1.092 | 3.555 | 1.179 |
| 13 | 22.5 | 3.887 | 1.092 | 3.674 | 1.218 |
| 14 | 22.5 | 3.891 | 1.092 | 3.073 | 1.019 |
| 15 | 22.5 | 3.995 | 1.092 | 3.726 | 1.236 |
| 16 | 22.5 | 4.623 | 1.092 | 4.579 | 1.519 |
| 17 | 22.5 | 5.148 | 1.092 | 4.419 | 1.465 |
| 18 | 22.5 | 5.210 | 1.092 | 5.193 | 1.722 |
| 19 | 45.0 | 3.712 | 0.975 | 3.500 | 1.036 |
| 20 | 45.0 | 3.902 | 0.975 | 3.877 | 1.148 |
| 21 | 45.0 | 3.948 | 0.975 | 3.821 | 1.131 |
| 22 | 45.0 | 4.397 | 0.975 | 4.579 | 1.355 |
| 23 | 45.0 | 4.402 | 0.975 | 4.763 | 1.410 |
| 24 | 45.0 | 4.457 | 0.975 | 4.844 | 1.434 |
| 25 | 45.0 | 4.535 | 0.975 | 4.648 | 1.376 |
| 26 | 45.0 | 4.617 | 0.975 | 4.896 | 1.449 |
| 27 | 45.0 | 5.326 | 0.975 | 5.271 | 1.560 |
| 28 | 67.5 | 3.971 | 0.874 | 4.006 | 1.063 |
| 29 | 67.5 | 3.987 | 0.874 | 3.780 | 1.003 |
| 30 | 67.5 | 3.998 | 0.874 | 3.792 | 1.006 |
| 31 | 67.5 | 4.504 | 0.874 | 4.941 | 1.311 |
| 32 | 67.5 | 4.636 | 0.874 | 4.447 | 1.180 |
| 33 | 67.5 | 4.642 | 0.874 | 5.021 | 1.332 |
| 34 | 67.5 | 4.653 | 0.874 | 4.745 | 1.259 |
| 35 | 67.5 | 4.664 | 0.874 | 4.745 | 1.259 |
| 36 | 67.5 | 5.313 | 0.874 | 5.250 | 1.393 |
| 37 | 67.5 | 5.334 | 0.874 | 5.116 | 1.358 |
| 38 | 90.0 | 3.967 | 0.823 | 4.082 | 1.021 |
| 39 | 90.0 | 3.978 | 0.823 | 4.154 | 1.039 |
| 40 | 90.0 | 4.004 | 0.823 | 4.262 | 1.066 |
| 41 | 90.0 | 4.599 | 0.823 | 4.958 | 1.240 |
| 42 | 90.0 | 4.651 | 0.823 | 5.055 | 1.264 |
| 43 | 90.0 | 5.285 | 0.823 | 5.320 | 1.330 |
| 44 | 90.0 | 5.401 | 0.823 | 5.202 | 1.301 |
