# Supplementary material for: Dynamic fracture mechanics and energy distribution rate response characteristics of coal containing bedding structure
Source: PLoS One. 2021 Jun 24;16(6):e0247908. doi: 10.1371/journal.pone.0247908 (PMC8224884; doi:10.1371/journal.pone.0247908)
Supplement: S4 Table — (DOCX) [file pone.0247908.s004.docx]

Table 4 Results of dynamic fracture toughness of coal samples (*α_a_* = 0.28).

| Specimen number | Bedding angle  (^o^) | Loading velocity (m·s^-1^) | Dimensionless stress intensity factor (1) | Maximum load  (KN) | Fracture toughness (MPa·m^1/2^) |
| --- | --- | --- | --- | --- | --- |
| 1 | 0.0 | 3.879 | 1.644 | 2.832 | 1.413 |
| 2 | 0.0 | 4.331 | 1.644 | 2.832 | 1.413 |
| 3 | 0.0 | 4.554 | 1.644 | 3.459 | 1.726 |
| 4 | 0.0 | 4.597 | 1.644 | 3.045 | 1.520 |
| 5 | 0.0 | 4.589 | 1.644 | 3.486 | 1.740 |
| 6 | 0.0 | 5.242 | 1.644 | 3.906 | 1.949 |
| 7 | 0.0 | 5.232 | 1.644 | 3.939 | 1.966 |
| 8 | 0.0 | 5.252 | 1.644 | 3.968 | 1.980 |
| 9 | 0.0 | 5.359 | 1.644 | 3.843 | 1.918 |
| 10 | 22.5 | 3.740 | 1.469 | 2.937 | 1.310 |
| 11 | 22.5 | 4.537 | 1.469 | 3.563 | 1.590 |
| 12 | 22.5 | 5.262 | 1.469 | 3.843 | 1.714 |
| 13 | 45.0 | 3.591 | 1.291 | 2.823 | 1.107 |
| 14 | 45.0 | 3.683 | 1.291 | 2.826 | 1.108 |
| 15 | 45.0 | 3.828 | 1.291 | 2.844 | 1.115 |
| 16 | 45.0 | 4.162 | 1.291 | 3.192 | 1.252 |
| 17 | 45.0 | 4.538 | 1.291 | 2.697 | 1.057 |
| 18 | 45.0 | 4.569 | 1.291 | 3.646 | 1.429 |
| 19 | 45.0 | 4.603 | 1.291 | 3.681 | 1.443 |
| 20 | 45.0 | 4.667 | 1.291 | 3.605 | 1.413 |
| 21 | 45.0 | 4.681 | 1.291 | 3.718 | 1.458 |
| 22 | 45.0 | 4.691 | 1.291 | 3.646 | 1.429 |
| 23 | 45.0 | 4.839 | 1.291 | 3.934 | 1.542 |
| 24 | 45.0 | 5.275 | 1.291 | 3.934 | 1.542 |
| 25 | 67.5 | 3.827 | 1.141 | 2.956 | 1.024 |
| 26 | 67.5 | 3.833 | 1.141 | 2.855 | 0.989 |
| 27 | 67.5 | 3.847 | 1.141 | 2.992 | 1.037 |
| 28 | 67.5 | 3.987 | 1.141 | 3.025 | 1.048 |
| 29 | 67.5 | 4.513 | 1.141 | 3.673 | 1.273 |
| 30 | 67.5 | 4.527 | 1.141 | 3.635 | 1.260 |
| 31 | 67.5 | 4.543 | 1.141 | 3.493 | 1.210 |
| 32 | 67.5 | 4.676 | 1.141 | 3.683 | 1.276 |
| 33 | 67.5 | 4.688 | 1.141 | 3.427 | 1.188 |
| 34 | 67.5 | 5.227 | 1.141 | 3.934 | 1.363 |
| 35 | 67.5 | 5.303 | 1.141 | 3.935 | 1.363 |
| 36 | 67.5 | 6.200 | 1.141 | 4.055 | 1.405 |
| 37 | 90.0 | 3.931 | 1.064 | 3.034 | 0.980 |
| 38 | 90.0 | 3.943 | 1.064 | 3.092 | 0.999 |
| 39 | 90.0 | 3.982 | 1.064 | 3.129 | 1.010 |
| 40 | 90.0 | 4.009 | 1.064 | 3.058 | 0.988 |
| 41 | 90.0 | 4.127 | 1.064 | 3.395 | 1.097 |
| 42 | 90.0 | 4.361 | 1.064 | 3.639 | 1.175 |
| 43 | 90.0 | 4.507 | 1.064 | 3.821 | 1.234 |
| 44 | 90.0 | 5.279 | 1.064 | 3.990 | 1.289 |
