# Supplementary material for: Dynamic fracture mechanics and energy distribution rate response characteristics of coal containing bedding structure
Source: PLoS One. 2021 Jun 24;16(6):e0247908. doi: 10.1371/journal.pone.0247908 (PMC8224884; doi:10.1371/journal.pone.0247908)
Supplement: S5 Table — (DOCX) [file pone.0247908.s005.docx]

Table 5 Results of dynamic fracture toughness of coal samples (*α_a_* = 0.40).

| Specimen number | Bedding angle  (^o^) | Loading velocity (m·s^-1^) | Dimensionless stress intensity factor (1) | Maximum load  (KN) | Fracture toughness (MPa·m^1/2^) |
| --- | --- | --- | --- | --- | --- |
| 1 | 0.0 | 3.838 | 2.252 | 2.072 | 1.416 |
| 2 | 0.0 | 3.945 | 2.252 | 2.080 | 1.422 |
| 3 | 0.0 | 4.502 | 2.252 | 2.659 | 1.818 |
| 4 | 0.0 | 4.476 | 2.252 | 2.659 | 1.818 |
| 5 | 0.0 | 5.212 | 2.252 | 2.881 | 1.970 |
| 6 | 0.0 | 5.232 | 2.252 | 2.881 | 1.970 |
| 7 | 0.0 | 5.252 | 2.252 | 2.908 | 1.988 |
| 8 | 0.0 | 5.651 | 2.252 | 2.926 | 2.001 |
| 9 | 22.5 | 3.859 | 2.014 | 2.109 | 1.289 |
| 10 | 22.5 | 3.878 | 2.014 | 1.819 | 1.112 |
| 11 | 22.5 | 3.909 | 2.014 | 2.100 | 1.284 |
| 12 | 22.5 | 4.517 | 2.014 | 2.694 | 1.647 |
| 13 | 22.5 | 4.585 | 2.014 | 2.654 | 1.623 |
| 14 | 22.5 | 5.285 | 2.014 | 2.909 | 1.778 |
| 15 | 22.5 | 5.341 | 2.014 | 2.909 | 1.778 |
| 16 | 22.5 | 5.652 | 2.014 | 2.787 | 1.704 |
| 17 | 45.0 | 3.788 | 1.771 | 2.151 | 1.156 |
| 18 | 45.0 | 3.968 | 1.771 | 2.225 | 1.196 |
| 19 | 45.0 | 4.476 | 1.771 | 2.672 | 1.437 |
| 20 | 45.0 | 4.502 | 1.771 | 2.680 | 1.441 |
| 21 | 45.0 | 4.598 | 1.771 | 2.486 | 1.336 |
| 22 | 45.0 | 5.232 | 1.771 | 2.891 | 1.554 |
| 23 | 45.0 | 5.305 | 1.771 | 2.849 | 1.532 |
| 24 | 45.0 | 5.391 | 1.771 | 2.868 | 1.542 |
| 25 | 67.5 | 3.714 | 1.532 | 2.102 | 0.978 |
| 26 | 67.5 | 3.867 | 1.532 | 2.201 | 1.024 |
| 27 | 67.5 | 4.545 | 1.532 | 2.712 | 1.262 |
| 28 | 67.5 | 4.507 | 1.532 | 2.740 | 1.275 |
| 29 | 67.5 | 4.592 | 1.532 | 2.543 | 1.183 |
| 30 | 67.5 | 5.148 | 1.532 | 2.704 | 1.258 |
| 31 | 67.5 | 5.292 | 1.532 | 2.727 | 1.269 |
| 32 | 67.5 | 5.369 | 1.532 | 2.893 | 1.346 |
| 33 | 67.5 | 5.511 | 1.532 | 2.864 | 1.332 |
| 34 | 90.0 | 3.811 | 1.466 | 2.206 | 0.982 |
| 35 | 90.0 | 3.878 | 1.466 | 2.238 | 0.996 |
| 36 | 90.0 | 3.945 | 1.466 | 2.283 | 1.016 |
| 37 | 90.0 | 4.282 | 1.466 | 2.488 | 1.108 |
| 38 | 90.0 | 4.252 | 1.466 | 2.507 | 1.116 |
| 39 | 90.0 | 4.647 | 1.466 | 2.760 | 1.229 |
| 40 | 90.0 | 5.261 | 1.466 | 2.904 | 1.293 |
| 41 | 90.0 | 5.327 | 1.466 | 2.751 | 1.225 |
