# Supplementary material for: Dynamic fracture mechanics and energy distribution rate response characteristics of coal containing bedding structure
Source: PLoS One. 2021 Jun 24;16(6):e0247908. doi: 10.1371/journal.pone.0247908 (PMC8224884; doi:10.1371/journal.pone.0247908)
Supplement: S6 Table — (DOCX) [file pone.0247908.s006.docx]

S6 Table. Energy dissipation statistics of coal samples.

| Bedding angle (°) | Impact speed (m·s^-1^) | Incident energy (J) | | Absorption energy (J) | Impact rod kinetic energy (J) | Fracture energy (J) | Residual kinetic energy (J) | Energy dissipation rate (%) |
| --- | --- | --- | --- | --- | --- | --- | --- | --- |
| 0.0 | 4.826_11_±0.54 | 12.870_11_±4.41 | 1.984_11_±0.48 | | 72.14_11_±15.6 | 1.408_11_±0.44 | 0.576_11_±0.24 | 2.04_11_±0.69 |
| 22.5 | 4.324_11_±0.58 | 5.137_11_±2.32 | | 0.936_11_±0.46 | 58.17_11_±16.1 | 0.748_11_±0.39 | 0.188_11_±0.09 | 1.33_11_±0.72 |
| 45.0 | 4.370_10_±0.59 | 6.741_10_±2.62 | | 1.667_10_±0.65 | 59.44_10_±16.2 | 1.311_10_±0.55 | 0.356_10_±0.22 | 2.24_10_±0.91 |
| 67.5 | 4.114_6_±0.37 | 5.755_6_±0.85 | | 1.521_6_±0.75 | 52.15_6_±9.5 | 1.202_6_±0.72 | 0.319_6_±0.11 | 2.29_6_±1.42 |
| 90.0 | 4.308_16_±0.47 | 5.468_16_±1.99 | | 1.441_16_±0.66 | 57.47_16_±13.3 | 1.164_16_±0.61 | 0.277_16_±0.14 | 2.08_16_±1.19 |

*Note: The data in the table are expressed in the form of "Average value _Number of samples_* ± *Standard deviation".*
